# Fronto-Striatal Atrophy in Behavioral Variant Frontotemporal Dementia and Alzheimer’s Disease

**DOI:** 10.3389/fneur.2015.00147

**Published:** 2015-07-01

**Authors:** Maxime Bertoux, Claire O’Callaghan, Emma Flanagan, John R. Hodges, Michael Hornberger

**Affiliations:** ^1^Neurosciences Research Australia (NeuRA), Randwick, NSW, Australia; ^2^Department of Clinical Neurosciences, University of Cambridge, Cambridge, UK; ^3^School of Medical Sciences, University of New South Wales, Sydney, NSW, Australia

**Keywords:** frontotemporal dementia, Alzheimer’s disease, striatum, fronto-striatal circuits, ventromedial prefrontal cortex

## Abstract

Behavioral variant frontotemporal dementia (bvFTD) has only recently been associated with significant striatal atrophy, whereas the striatum appears to be relatively preserved in Alzheimer’s disease (AD). Considering the critical role the striatum has in cognition and behavior, striatal degeneration, together with frontal atrophy, could be responsible of some characteristic symptoms in bvFTD and emerges therefore as promising novel diagnostic biomarker to distinguish bvFTD and AD. Previous studies have, however, only taken either cortical or striatal atrophy into account when comparing the two diseases. In this study, we establish for the first time a profile of fronto-striatal atrophy in 23 bvFTD and 29 AD patients at presentation, based on the structural connectivity of striatal and cortical regions. Patients are compared to 50 healthy controls by using a novel probabilistic connectivity atlas, which defines striatal regions by their cortical white-matter connectivity, allowing us to explore the degeneration of the frontal and striatal regions that are functionally linked. Comparisons with controls revealed that bvFTD showed substantial fronto-striatal atrophy affecting the ventral as well as anterior and posterior dorso-lateral prefrontal cortices and the related striatal subregions. In contrast, AD showed few fronto-striatal atrophy, despite having significant posterior dorso-lateral prefrontal degeneration. Direct comparison between bvFTD and AD revealed significantly more atrophy in the ventral striatal–ventromedial prefrontal cortex regions in bvFTD. Consequently, deficits in ventral fronto-striatal regions emerge as promising novel and efficient diagnosis biomarker for bvFTD. Future investigations into the contributions of these fronto-striatal loops on bvFTD symptomology are needed to develop simple diagnostic and disease tracking algorithms.

## Introduction

Behavioral variant frontotemporal dementia (bvFTD) is the most common subtype of frontotemporal dementia (FTD), and characterized by deterioration of behavior and cognition, including impaired social interactions, disinhibition, apathy, or impairment in adaptive functioning, in association with prominent frontal and temporal lobar atrophy (1).

Imaging studies have described extensively the cortical atrophy that occurs in bvFTD, such as the characteristic progressive atrophy in frontal and polar temporal lobe brain regions (2, 3). In particular, atrophy in the ventromedial prefrontal cortex (VMPFC) has been shown to be specific to bvFTD, as it is already affected in the early disease stages (4, 5). Nevertheless, prefrontal, including VMPFC, atrophy is also apparent in many Alzheimer’s disease (AD) patients (6–8) and thus a sole reliance on VMPFC atrophy might lack diagnostic specificity to distinguish bvFTD and AD (7).

Recent anatomical and neuropathological studies have shown that subcortical regions are also affected in FTD. Among these subcortical structures, there is mounting evidence that the striatum is affected particularly early and significantly (4, 9–11). Most consistently, bvFTD show significant cell loss across the entire striatum (i.e., nucleus accumbens, caudate nucleus, and putamen) relative to controls and AD (12–14). In contrast, AD striatal atrophy studies have reported either no change or only subtle atrophy in the caudate, which has been taken to be proportional to the whole-brain atrophy that occurs with disease progression (12, 13, 15). Surprisingly, however, striatal atrophy has not been considered as a diagnostic biomarker for bvFTD so far, even though it shows a high-disease sensitivity and specificity (16, 17).

The striatum has been recognized to act as a critical nexus in the brain as it receives afferents and efferents from multiple cortical and subcortical regions and is part of affective/limbic, cognitive, and motor brain circuits (18, 19). Recent functional neuroimaging evidence in controls suggests that the functionality of the striatum does not map onto the discrete anatomical regions (nucleus accumbens, caudate nucleus, and putamen), instead the cortico-striatal connectivity is more promising in mapping cognitive functions to striatal regions comprising multiple anatomical subregions. Indeed, reward-related cognition, such as reward valuation or anticipation, has been associated with both ventral striatal and VMPFC activations in fMRI studies (20). Similarly, dorsal striatal and motor cortex activation has been associated with motor planning and execution processes (21, 22). Thus, mapping the cortico-striatal and particularly fronto-striatal atrophy based on their connectivity instead of anatomical boundaries emerges as important in understanding the diagnostic utility of striatal atrophy in bvFTD and AD. Indeed, combined atrophy of striatal and frontal regions might provide better neuroimaging biomarkers for bvFTD pathology than each region in isolation.

The aim of the current study is to establish a profile of fronto-striatal atrophy in bvFTD and AD at clinical presentation. Instead of exploring the structural connectivity between the frontal cortex and the striatum, or using anatomically defined discrete striatal regions (nucleus accumbens, caudate nucleus, and putamen), as previous studies have done, we employed a novel probabilistic connectivity striatal atlas [Oxford-GSK-Imanova Striatal Connectity Atlas (23)], which defines striatal regions by their cortical white-matter connectivity. Thus, the atlas enables identification of cortical and substriatal regions that are structurally linked and have therefore likely shared functions. Using this novel approach based on both functionality and anatomy, we hypothesized that bvFTD and AD would show distinct patterns of fronto-striatal atrophy, with bvFTD showing more VMPFC and ventral striatal atrophy while AD would should few striatal atrophy despite having PFC atrophy.

## Materials and Methods

### Case selection

Patients were selected from the Frontotemporal Dementia Research Group (FRONTIER) at Neuroscience Research Australia (NeuRA) following study approval by the University of New South Wales Human research Ethics Advisory Panel D (Biomedical, ref. 100035). Every participant signed an informed consent and the study was in compliance with the Declaration of Helsinki. It resulted in a sample of 23 bvFTD, 29 AD patients, and 50 controls. All bvFTD patients met current consensus diagnosis criteria (24). In light of the recent recognition of the phenocopy syndrome (25) only bvFTD patients with evidence of clear decline as reported by the caregivers and atrophy on MRI scans were included in the study. All AD patients met NINCNS-ADRDA diagnostic criteria (26) for probable AD (see Table 1 for demographic details). Healthy controls were selected from a healthy volunteer panel or were spouses/carers of patients.

**Table 1:** **Demographics, behavioral, and neuropsychological screening data for bvFTD, AD, and controls**.

Demographics, behavioral and cognitive tests	bvFTD	AD	Controls
*N*	23	29	50
Sex (M/F)	15/8	18/11	22/28
Mean age (years)	60.9 (9.8)*	65.0 (8.1)	68.8 (6.3)
Duration of disease (years)	3.7 (2.4)^†^	2.8 (1.0)^†^	N.A.
FRS	−1.3 (1.6)^†^	0.8 (1.5)^†^	N.A.
CBI (total score)	72.5 (33.7)^†^^,^*	38.1 (22.9)^†^^,^*	7.1 (8.1)
ACE-R (total score)	73.6 (15.8)*	77.1 (9.7)*	94.6 (3.6)

*p < 0.001 compared to controls.

*^†^*p* < 0.001 when comparing patients groups*.

*Mean (SD)*.

### Clinical assessment

All patients underwent the frontotemporal dementia rating scale [FRS (27)], a clinical scale based on carers’ interview aiming to assess disease severity; the Cambridge behavioral inventory [CBI (28)] to evaluates behavioral symptoms and the Addenbrooke’s cognitive examination revised [ACE-R (29)] as a measure of cognitive efficiency.

### Imaging acquisition

All patient and controls underwent the same imaging protocol with whole-brain T1 using a 3-T Philips MRI scanner with standard quadrature head coil (eight channels). The 3D T1-weighted sequences were acquired as follows: coronal orientation, 256 × 256 matrix, 200 slices, 1 mm × 1 mm in-plane resolution, slice thickness 1 mm, TE/TR = 2.6/5.8 ms.

### Voxel based morphometry analysis: Pre-processing

3D T1-weighted sequences were analyzed with FSL-voxel based morphometry (VBM), a VBM analysis (30, 31), which is part of the FSL software package (http://www.fmrib.ox.ac.uk/fsl/fslvbm/index.html) (32). First, tissue segmentation was carried out using FMRIB’s automatic segmentation tool (FAST) (33) from brain-extracted images. The resulting gray-matter partial volume maps were then aligned to the Montreal Neurological Institute standard space (MNI152) using the non-linear registration approach using FNIRT (34, 35), which uses a b-spline representation of the registration warp field (36). Default settings were used for these steps, but quality control for each scan was performed and slight alteration of the search space for the segmentation algorithm was performed for some patients with severe atrophy. The registered partial volume maps were then modulated (to correct for local expansion or contraction) by dividing them by the Jacobian of the warp field. Importantly, the Jacobian modulation step did not include the affine part of the registration, which means that the data are normalized for head size as a scaling effect (37). The modulated images were then smoothed with an isotropic Gaussian kernel with a SD of 2 mm (FWHM: 5 mm).

### Voxel based morphometry analysis: ROI analyses

Instead of using arbitrary anatomical landmarks to subdivide the striatum, we use substriatal ROI from the Oxford-GSK-Imanova Striatal Connectity Atlas (23), a probabilistic atlas of substriatal regions segmented according to their white-matter connectivity to cortical regions. We selected three striatal ROIs, designed according to their prefrontal connexions, each being associated with a particular anatomical region within the frontal lobe: the ventromedial PFC (VMPFC), the anterior dorso-lateral PFC (A-DLPFC), and posterior dorso-lateral PFC (P-DLPFC). As a complement of this analysis, we also investigated the atrophy in each related prefrontal ROIs.

#### Striatum VMPFC-Connected ROI

This striatal subregion was defined according to the projections from the VMPFC (include the projections from anterior, medial, and posterior orbital gyri, from the gyrus rectus and from the subcallosal gyrus – together composing the VMPFC ROI).

#### Striatum A-DLPFC-Connected and A-DLPFC ROI

This striatal region was defined according to the projections from the anterior DLPFC (which include the projections of the rostral superior and middle frontal gyri and from the dorsal prefrontal cortex – together composing the A-DLPFC ROI).

#### Striatum P-DLPFC-Connected and P-DLPFC ROI

This ROI was defined according to the projections from the more posterior dorso-lateral regions of the frontal lobe, without the precentral gyrus (including the caudal portions of lateral and medial superior gyrus as well as the caudal middle and caudal inferior frontal gyri – together composing the P-DLPFC).

The three groups were contrasted (1) for each striatal ROI (striatal analysis) and (2) for each prefrontal ROI (prefrontal analysis). A voxelwise general linear model (GLM) was applied and permutation-based non-parametric testing was used to form clusters with the threshold-free cluster enhancement (TFCE) method (35), tested for significance at *p* < 0.05, corrected for multiple comparisons via family-wise error (FWE) correction across space. Age and disease severity as measured by the FRS (27) was included as covariates in all analyses. Sex was not included as a covariate because it did have no effect on the variables of interest.

Finally, in patient groups, the mean values of gray-matter intensity were extracted from the result maps given by the contrast with the control group. The extraction of these values was performed for each ROI, for the results of both striatal (striatum-VMPFC connected, striatum-A-DLPFC connected, striatum-P-DLPFC connected) and cortical analyses (VMPFC, A-DLPFC, P-DLPFC). Striatal and prefrontal mean gray-matter intensity values were then correlated to investigate the relationship of striatal and prefrontal atrophy within bvFTD and AD.

### Statistics

Using SPSS 20 (SPSS, Chicago, IL, USA), one-way ANOVA were conducted to compare demographic and background neuropsychological data cross groups, followed by Tukey *post hoc* tests. Variables were plotted and checked for normality of distribution by Kolmogorov–Smirnov tests. Variables revealing non-normal distributions were log transformed and the appropriate log values were used in the analyses. Correlations were explored with Pearson coefficient and were corrected for multiple comparison (Bonferroni’s correction).

## Results

### Demographics, behavioral, and cognitive screening measures

Participants did not differ significantly on education or gender distribution; however, controls were significantly older than bvFTD (*p* < 0.001) but not AD (*p* > 0.1) (Table 1). bvFTD patients had also a higher disease severity as measured by the FRS compared to AD (*p* < 0.001).

On the behavioral screening test (CBI), bvFTD patients performed worse than AD and both patient groups were impaired compared to controls (all *p* values <0.001). On general cognitive screening, controls performed significantly better than both patient groups (*p* < 0.001), whereas bvFTD and AD did not differ significantly from each other.

### VBM – Group analyses

#### Striatum Analysis

##### Striatum VMPFC-connected ROI

**Figure 1:**
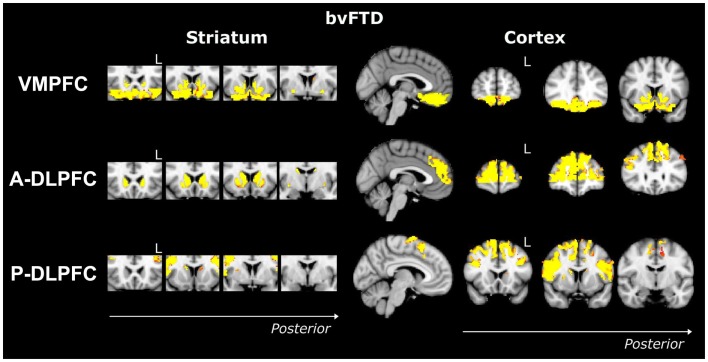
**Voxel-based morphometry analyses showing areas that were atrophic in bvFTD compared to controls for the three ROIs (VMPFC, A-DLPFC, and P-DLPFC), in striatum (left) and cortex (right)**. Clusters are overlaid on the MNI standard brain. Colored voxels show regions that were significant in the analyses for *p* < 0.05 corrected for multiple comparisons via family-wise error (FWE) and a voxel threshold of 20 contiguous voxels. Images follow radiological convention (left is right and right is left) and “L” indicates the left for coronal slices.

**Figure 2:**
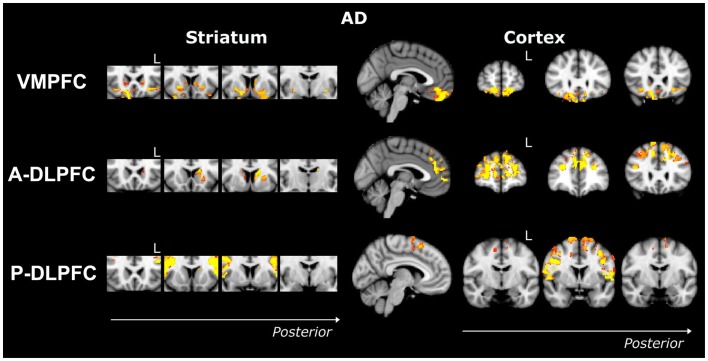
**Voxel-based morphometry analyses showing areas that were atrophic in AD compared to controls for three ROIs (VMPFC, A-DLPFC, and P-DLPFC), in striatum (left) and cortex (right)**. Clusters are overlaid on the MNI standard brain. Colored voxels show regions that were significant in the analyses for *p* < 0.05 corrected for multiple comparisons via family-wise error (FWE) and a voxel threshold of 20 contiguous voxels. Images follow radiological convention (left is right and right is left) and “L” indicates the left for coronal slices.

**Figure 3:**
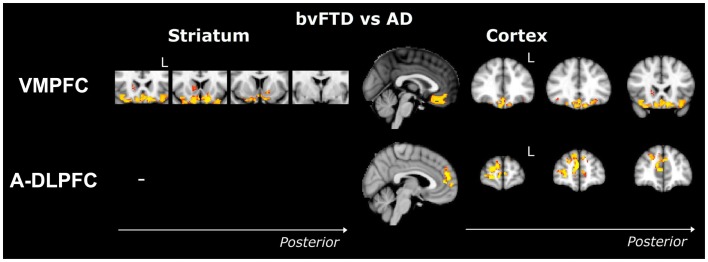
**Voxel-based morphometry analyses showing areas that were atrophic in bvFTD compared to AD**. Results were only found in VMPFC and A-DLPFC ROIs. Clusters are overlaid on the MNI standard brain. Colored voxels show regions that were significant in the analyses for *p* < 0.05 corrected for multiple comparisons via Family-wise Error (FWE) and a voxel threshold of 20 contiguous voxels. Images follow radiological convention (left is right and right is left) and “L” indicates the left for coronal slices.

Compared to controls, bvFTD patients showed widespread striatal atrophy, including bilateral nucleus accumbens, caudate nucleus, and putamen (Figure 1). Compared to controls, Alzheimer patients (Figure 2) showed a striatal atrophy that encompassed bilateral caudate nucleus and putamen. When contrasting directly bvFTD with AD patients, analysis revealed striatal atrophy in the right anterior caudate nucleus (Figure 3).

##### Striatum A-DLPFC-connected ROI

Compared to controls, bvFTD patients presented with a striatal atrophy that included bilateral nuclei accumbens, caudate nuclei, pre-commissural putamen, and, to a lesser degree, post-commissural putamen. Compared to controls, AD showed an atrophy of the striatum characterized by bilateral atrophy of the caudate nucleus but prominent in the left side where it extended to the pre-commissural putamen. No differences were observed when contrasting bvFTD and AD directly.

##### Striatum P-DLPFC-connected ROI

Contrasted to controls, bvFTD presented with a striatal atrophy that involved bilateral caudate nuclei as well as right putamen, where degeneration was prominent in its pre-commissural part. Compared to controls, Alzheimer patients showed less pronounced striatal atrophy than bvFTD, which was circumscribed to the left caudate nucleus. No significant voxels were found when contrasting bvFTD with AD directly.

#### Prefrontal Analysis

##### VMPFC ROI

Contrasted to controls, bvFTD showed a bilateral and widespread atrophy of the ventral part of the frontal cortex, including lateral orbito-frontal cortex and median areas from the subcallosal gyrus to the median frontal pole. AD patients showed a bilateral atrophy of the ventro-median and lateral prefrontal cortices. When contrasting bvFTD with AD, bvFTD showed a greater VMPFC atrophy, circumscribed to the bilateral ventral median frontal cortex and subcallosal/paracingulate cortices, with rostral areas being relatively spared, but extended laterally to the inferior frontal gyrus.

##### A-DLPFC ROI

Compared to controls, bvFTD patients presented with a wide and bilateral cortical atrophy within the median and lateral prefrontal areas that included the paracingulate gyrus and the median part of the superior frontal gyrus, the middle frontal gyrus and the dorsal frontal pole. AD also presented with cortical atrophy that involved the same regions that in bvFTD, but to a lesser degree. When contrasting bvFTD to AD, atrophy was found in the bilateral superior gyrus, the frontal pole, and in the paracingulate gyrus, with a strong predominance in the right lobe.

##### P-DLPFC ROI

Contrasted to controls, the dlPFC in bvFTD was characterized by a bilateral atrophy of the precentral gyrus, extended to the caudal portions of the superior frontal gyrus and the opercular cortex as well as to the supplementary motor cortex. In AD, the same pattern of cortical atrophy was observed, but again to a lesser degree than bvFTD. No significant voxels were found when contrasting bvFTD with AD directly.

### Relationship between striatal and prefrontal atrophy

**Figure 4:**
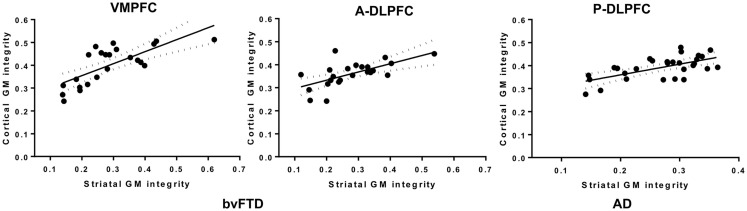
**Plots of the correlation between the mean individual grey-matter intensity values extracted from the striatum- VMPFC-connected and the striatum-A-DLPFC-connected and in the VMPFC and A-DLPFC in bvFTD patients (left frame) and from the striatum-P-DLPFC-connected and P-DLPFC in AD patients (right frame)**.

In the bvFTD group, the atrophy of the striatal subregions connected to the VMPFC was highly related (*R* = 0.81; *p* < 10^−6^) to the atrophy of the VMPFC (Figure 4). A significant correlation was also observed for the A-DLPFC (0.63; *p* < 0.001) and its connected striatal subregions, but not for the P-DLPFC (*R* = 0.31, NS). In the AD group, the atrophy in the VMPFC and in its connected striatal region was not correlated (*R* = 0.23, NS), as well as in the A-DLPFC and in its striatal subregions (*R* = 0.32, NS). In contrast, the atrophy in the P-DLPFC and in its striatal-connected region was significantly correlated (*R* = 0.66; *p* < 10^−4^).

## Discussion

Using a novel probabilistic connectivity atlas, we demonstrated different patterns of fronto-striatal atrophy in bvFTD and AD based on the structural connectivity of prefrontal and striatal regions (Table 2). More specifically, bvFTD patients showed significant striatal and global prefrontal atrophy, whereas AD patients showed only minor striatal atrophy while having significant dorso-lateral prefrontal atrophy. In particular, striatal regions connecting VMPFC cortex areas, including the ventral striatum were severely affected in bvFTD compared to AD. More importantly, we found clear correlations between striatal and cortical regions, which dissociated for bvFTD and AD, with bvFTD showing only atrophy correlations between VMPFC and related striatal regions as well as between anterior DLPFC and connected striatal regions, while AD showing only atrophy correlations between posterior DLPFC and related striatal regions. This suggests for the first time that only some of the striatal atrophy in bvFTD and AD is related to specific cortical atrophy in both diseases.

**Table 2:** **Schematic notation of the results in the striatal or frontal regions for the three different imaging contrasts**.

	Striatal regions connected to			Frontal regions		
	VMPFC	A-DLPFC	P-DLPFC	VMPFC	A-DLPFC	P-DLPFC
bvFTD vs. controls	++	+++	++	++++	++++	++++
AD vs. controls	+	++	+	+++	+++	+++
bvFTD vs. AD	+			+++	+++	

*+ Indicates the presence of atrophy*.

*+ > 100 voxels; +++ > 500 voxels; ++++ > 1000 voxels; ++++ > 5000 voxels*.

In more detail, we replicated the well-known prefrontal atrophy in bvFTD, affecting ventral, rostral, and dorsal regions within the PFC (2, 3). We observed a prefrontal atrophy in AD as well, affecting every region within the PFC but to a lesser degree compared to bvFTD, and especially in the VMPFC. We also replicated (Figure 1) previous striatal degeneration results in bvFTD with significant widespread striatal atrophy evident at presentation (4, 9, 10, 12, 13). Similarly, we also found a subtle atrophy (Figure 2) in the striatum for AD, in particular, in the caudate nucleus and putamen, thus replicating previous studies (7, 38, 39).

The striato-prefrontal atrophy found in AD might at first sight surprising, as AD is commonly characterized by temporal and parietal atrophy (40) although diffuse atrophy was observed in AD (41), involving frontal regions as well. One possible explanation of these results is that the striatal subregions covary with the general cortical atrophy seen in AD. Striatal atrophy in AD would thus occur as a consequence of the cortical atrophy (15), i.e., a knock-on effect with disease duration. Different disease mechanisms could be then responsible for striatal atrophy in AD and bvFTD. Indeed, while significant caudate atrophy, together with thalamus, has been recently reported in familial AD (42), studies in sporadic AD only reported a subtle caudate volume loss, quantified to be around 6–7% compared with age-matched control (38), although a different profile may be observed according to the age-of-onset and the apolipoprotein E genotype, as recently suggested (43). In contrast, bvFTD have been reported to show a 25% caudate volume reduction (44). Differences between sporadic and familial AD suggest that while the same cortico-subcortical network atrophy is involved in both forms of AD, each of the nodes of this circuit are differentially involved in the two respective degeneration processes (42).

In contrast, in bvFTD, the atrophy of the striatum could be concomitant to the cortical atrophy. Indeed, while striatal degeneration could be considered as a consequence of the frontal atrophy occurring early in the disease course, it is likely that the crucial importance of the connection between frontal lobes and striatum has a direct impact on frontal atrophy as well, and this is supported by the strong correlation found between gray-matter loss in the VMPFC and the ventral striatum in bvFTD and not in AD. VMPFC and ventral striatum are richly interconnected (45) and both areas are involved in the degeneration pattern of bvFTD. While the striatal atrophy could be a consequence of VMPFC atrophy, one can hypothesize that VMPFC could, in turn, be affected by the striatal atrophy. Given the central place, the striatum has in fronto-striatal network (18), the trans-striatal atrophy in bvFTD could be a resultant of more general fronto-striatal network dysfunction. Along these lines, Looi and colleagues (16) hypothesized that a disruption of any component of the fronto-striatal circuitry could not only affect the functions of this network but also may have up- and downstream effects attributed to trans-synaptic neurodegeneration. Nevertheless, this still needs to be verified longitudinally, and it would be, in particular, important to contrast fronto-striatal with striatal connections to parietal and temporal cortical areas to see reveal different patterns of up and downstream synaptic changes.

Among all the striato-cortical regions investigated, the limbic network appeared to discriminate most clearly bvFTD and AD (Figure 3). This region includes ventromedial, subcallosal, and polar prefrontal cortices as well as the nucleus accumbens, anterior ventral caudate nucleus, and ventral putamen. Dysfunctions of the VMPFC in bvFTD are well known and the atrophy of this region is considered as a characteristic of bvFTD (2, 5, 46). Importantly, atrophy in this region has also been directly associated with specific symptoms in bvFTD, such as neuropsychiatric changes (47), loss of insight (48), inhibitory dysfunction (49, 50), and social cognition impairments (50). This has led to suggestions that frontal and particularly VMPFC specific changes are potentially powerful diagnostic markers for FTD pathology, even in bvFTD patients presenting with additional memory problems (51, 52). Similarly, atrophy of the ventral striatum has been previously described in bvFTD but not in AD (12, 13, 39, 41, 53), which is consistent with our results. Given the importance of the connections between VMPFC and ventral striatum (23, 45) and the role of this ventral circuit in reward-guided choice behavior (54), the conjunctive and respective investigations of the VMPFC and ventral striatum could represent promising ways to enhance the diagnosis of bvFTD. This dovetails nicely with previous findings, such as the association between disinhibition and fronto-striatal atrophy in bvFTD (17, 55), as well as other behavioral features, such as apathy, reduced empathy, and aberrant motor behavior (56, 57). Finally, some studies have also reported that atrophy of the striatum directly predicts executive, language, and psychomotor dysfunctions and poorer general cognition (10, 13). Taken together, these results suggest that numerous cognitive or behavioral measures could be utilized to detect striatal atrophy. Therefore, more specific delineation of the respective frontal and striatal contributions to cognition and behavior is needed in order to develop novel scales or tasks assessing pure striatal dysfunctions, which could be an early biomarker of bvFTD. For example, impaired probabilistic association learning has been found in FTD and related to striatal atrophy (58), as well as impulsivity related to delay discounting (59). These studies are encouraging but do not specifically delineate or dissociate striatal and cortical contributions, which should be explored in the future.

On a clinical level, the diagnosis of bvFTD remains challenging purely on clinical, cognitive, and behavioral biomarkers. The revised diagnostic criteria of bvFTD (24) now require evidence of frontal and/or temporal atrophy on imaging, in order to qualify for a diagnosis of probable bvFTD. Nevertheless, as outlined above, cortical markers of atrophy might be not as helpful in distinguishing bvFTD and AD, in particular, for dorso-lateral prefrontal changes, which can be associated with both conditions. In particular, a percentage of AD patients present with prefrontal deficits (60), which can make them appear clinically very similar to bvFTD (61). Although the replication of our findings is needed in larger group of patients, they indicate that striatal atrophy in combination with VMPFC atrophy should be much more promising in distinguishing those patients *in vivo*. Thus, the pure clinical and diagnostic focus on cortical changes in bvFTD and AD might have overshadowed the more important striatal changes that are mostly present in bvFTD. Employment of striatal atrophy measures should have much higher diagnostic specificity than any cortical atrophy measures when attempting to discriminate bvFTD and AD, even at presentation (16). Clearly, it would be very important to confirm this notion in pathologically confirmed cases of bvFTD and AD. Also, it would be clearly important to track cortico-striatal changes longitudinally to map the network atrophy over the disease course for both conditions.

These findings have important clinical and diagnostic implications for bvFTD as, to date, the substantial striatal degeneration occurring in the course of the disease is not taken into account. Previous investigations have only taken into account either cortical or striatal atrophy in bvFTD and AD, while the current study reveals prefrontal-striatal atrophy profiles across the diseases. This approach was made possible by a novel striato-cortical anatomical connectivity atlas derived from diffusion magnetic resonance imaging and probabilistic tractography. This novel method, taking into account functional links between brain structures in a VBM – or structural – analysis leads toward network atrophy profiles and away from region specific diagnostics, which may be simplistic. Further, structural connectivity data are grounded on anatomical diffusion data, which are an alternative to functional resting-state networks data.

## Conclusion

In summary, our results show different profiles of cortico-striatal network atrophy in bvFTD and AD. Notably, the limbic circuitry network (ventral striatum and median/ventro-median prefrontal cortex) seems to best distinguish between the groups, with bvFTD manifesting considerably greater atrophy in these regions. It is important to investigate whether white-matter tract DTI findings would give a similar result for the identified networks. Furthermore, despite the numerous and converging studies that have established the importance of the limbic fronto-striatal circuit in reward-cognition and behavior (62), to date, the role of the striatum in the generation of bvFTD symptoms is unclear and need to be addressed by future studies analyzing correlation between behavioral scales or cognitive testing and striatal subregions. Such studies should delineate frontal and striatal contributions to behavior. Taken together, our current findings could have major implications for future diagnostic guidelines of bvFTD as they will allow taking into account the striatal changes in FTD that have been so far overlooked in the diagnostic process and help in the distinction process between bvFTD and AD.

## Conflict of Interest Statement

Maxime Bertoux reports no conflicts of interest, no financial interests, and no disclosures. He receives Marie Skłodowska-Curie fellowship from the European Commission. Claire O’Callaghan reports no conflicts of interest, no financial interests, and no disclosures. Emma Flanagan reports no conflicts of interest, no financial interests, and no disclosures. John R. Hodges reports no conflicts of interest and no financial interests. He is editorial board member of Nature Reviews Neurology, Aphasiology, Cognitive Neuropsychiatry and Cognitive Neuropsychology; personal compensation: publishing royalties for Cognitive Assessment for Clinicians (Oxford University Press, 2007) and Frontotemporal Dementia Syndromes (Cambridge University Press, 2007); funding support: National Health and Medical Research Council of Australia. Michael Hornberger reports no conflicts of interest and no financial interests. He is editorial board member of the Journal of Alzheimer’s Disease, Dementia and Cognitive Geriatrics, and the American Journal of Neurodegeneration. He receives grants and fellowships from the Australian government funded Australian Research Council (ARC) and National Health and Medical Research Council (NHMRC).
